# Supramolecular dye nanoassemblies for advanced diagnostics and therapies

**DOI:** 10.1002/btm2.10652

**Published:** 2024-02-13

**Authors:** Pouria Ramezani, Stefaan C. De Smedt, Félix Sauvage

**Affiliations:** ^1^ Laboratory of General Biochemistry and Physical Pharmacy, Faculty of Pharmaceutical Sciences Ghent University Ghent Belgium

**Keywords:** dyes, fluorescence imaging, nanoparticles, photoacoustic imaging, photodynamic therapy, photothermal therapy, photoablation, stabilizers

## Abstract

Dyes have conventionally been used in medicine for staining cells, tissues, and organelles. Since these compounds are also known as photosensitizers (PSs) which exhibit photoresponsivity upon photon illumination, there is a high desire towards formulating these molecules into nanoparticles (NPs) to achieve improved delivery efficiency and enhanced stability for novel imaging and therapeutic applications. Furthermore, it has been shown that some of the photophysical properties of these molecules can be altered upon NP formation thereby playing a major role in the outcome of their application. In this review, we primarily focus on introducing dye categories, their formulation strategies and how these strategies affect their photophysical properties in the context of photothermal and non‐photothermal applications. More specifically, the most recent progress showing the potential of dye supramolecular assemblies in modalities such as photoacoustic and fluorescence imaging, photothermal and photodynamic therapies as well as their employment in photoablation as a novel modality will be outlined. Aside from their photophysical activity, we delve shortly into the emerging application of dyes as drug stabilizing agents where these molecules are used together with aggregator molecules to form stable nanoparticles.

**Translational Impact Statement**

In recent years, there has been a huge interest in applying dye molecules for diagnosis and therapy benefiting from nanotechnology. In this review, we highlight the benefits associated with employing these molecules using nanotechnology approaches. These benefits are backed up by *in vivo* data showing promise for further translation into the clinic.

## 1 INTRODUCTION

Dyes, are small organic molecules that impart color to materials by sticking to their surfaces.^1^ This unique property enables them to be applied for staining different materials such as, foods, drugs and cosmetics or live cells and tissue.^2^ All dyes share one characteristic: the ability to absorb light between the visible (380–700 nm) and near‐infrared (NIR; 700–1700 nm) regions.^3^ The absorption of light defined as discrete energy packets namely photons is measured by the absorption coefficient. This value is much higher in dyes compared to other molecules.^4^ Consequent to the absorption of light photons, an excitation of the valence electrons on dye molecules happens leading to movement of available electrons to a higher quantum energy state. Since these molecules are energetically unstable, excess energy is expressed by emission of light or heat or even the two simultaneously, endowing them with unique properties for biomedical imaging and therapy.^5^

Due to the interesting biomedical potential dyes possess, efforts have been made to optimize the administration of these compounds. For this purpose, utilizing them into nanoparticles (NPs) is attractive as these supramolecular assemblies usually enhance the delivery of various compounds to cells and tissue passively or actively and also reduce off target distribution aiming for diagnosis and therapy of many conditions such as cardiovascular diseases, central nervous system (CNS) diseases and cancers.^6^, ^7^, ^8^, ^9^

This review aims to elucidate how assembling these molecules into NPs has helped employ dyes for biomedical applications *in vivo*. The biomedical applications in question are diagnostic, therapeutic or theranostic and involve fluorescence and photoacoustic imaging as well as photodynamic and photothermal therapy. Additionally, two novel applications of these molecules are introduced: (a) photoablation in which undesired biological specimens or barriers are treated or removed and (b) stabilization of hydrophobic compounds where aggregator molecules are used jointly with dyes to form stable nanoparticles.

## 2 A BRIEF HISTORY OF DYES AND THEIR USE IN HISTOLOGY

Throughout the history of medicine, dyes have had a prominent role in histology and were used to highlight entities within the body, tissues or cells.^10^ Histological and biochemical studies at the microscopic level require visualizing cellular organelles, cells, and tissues to provide more contrast in order to distinguish areas that are intended to be visualized from the surroundings. In this regard, dyes display great potential considering their capacity to bind onto surfaces. Carmine was the first compound to be used for staining tissues and cells by Joseph Von Gerlach in 1850s making him the pioneer in the application of dyes for staining. In his experiments, he observed much more stainability in the nuclei of endothelial cells. Furthermore, he obtained stained specimens of the brain and spinal cord and could identify the reticular structure of the nervous system.^11^ In 1882, Hans Christian Gram gave his name to the gram staining methods which are based upon differentiation of bacteria depending on their cell wall content. The high level of peptidoglycan content in gram positive cell wall enables the crystal violet dye to form crystals which are resistant to decolorization by solvents such as ethanol and acetone whereas gram negative microorganisms own a higher level of lipids making the washing process of the stain easier upon applying decolorizer solvents.^12^ Up until this date, gram staining is still used for discriminating between gram positive and negative bacteria allowing their classification which is important in medicine for choosing proper antibiotic therapies. Hematoxylin–eosin (H&E) staining is also a conventional technique for tissue visualization to get structural information regarding cell morphology and tissue structure which enables diagnosis of conditions induced by tissue or cell abnormalities. This method has been widely employed in medicine for identification of several types of cancers (e.g., melanoma),^13^ inflammatory diseases (e.g., arteritis)^14^ and other pathological abnormalities secondary to chronic conditions (e.g., hypertensive nephropathy caused by chronic kidney disease).^15^ This procedure involves two stains, hematoxylin which colorizes the nucleus by imparting a deep blue‐purple color and eosin, which gives a purple color to the cytoplasm and the extracellular matrix by binding to different proteins. The golgi zone can be detected in areas where staining is absent since these compartments do not absorb the stains. Also, polyribosomes can be identified by a distinct blue cast in the cytoplasm. The major limitation of this type of staining is that it is not compatible with immunofluorescence assays.^16^ Some morphological and histological studies require the evaluation of the distribution of biological macromolecules in specimens. Lipids, a major biological component, are detected via sudan staining. Since these dyes show high affinity towards lipids, they can help visualize and quantify lipid structures; examples include sudan black b for staining myelin in the central nervous system^17^ or sudan red III for fecal fat detection.^18^ Carbohydrates can also be stained by the periodic‐acid shiff (PAS) method in which fatty vesicles in liver cells are visualized by highlighting the glycogen‐rich cells with fuchsine dye.^19^ All these conventional techniques have been in use for many years and have been improved by reducing the number of steps or changing the composition of dyes to enhance the contrast of the images of the specimen captured by light microscopy. These staining methods have led to a better understating of morphological changes at the tissue or even cell level improving diagnosis and helping medical decision. However, there are serious limitations in their application when it comes to studying real‐time nanoscale biological processes where staining is required to be as precise as possible at the molecular level and not to alter the viability of the cells.

## 3 FROM DYES TO PHOTOSENSITIZERS

### 3.1 Photosensitizers: their history and development

By the end of the 1970s, researchers began to grow interest in the application of dyes as photosensitizers (PSs) apart from their use for histological and biochemical purposes. PSs are molecules that have high light absorption coefficients in certain wavelengths. Their exposure to light causes the excitation of their available electrons. To relapse to the relaxed state, PSs dissipate the excess energy following different pathways that can be exploited in biomedical sciences.

PSs vary in composition and are not limited to small organic molecules. Organic nanomaterials such as carbon‐based (e.g., graphene and carbon nanotube) nanomaterials,^20^, ^21^ inorganic nanomaterials (e.g., gold and iron‐oxide nanoparticles)^22^, ^23^ and quantum dots consisting of heavy metals^24^, ^25^ are examples of photosensitive materials. Despite the fact that organic‐based nanomaterials consist of carbon, in vitro and in vivo toxicity studies have brought up some concerns. The majority of these materials consist of non‐degradable covalent bonds thus their degradation and clearance can generally be challenging for the human body.^26^ As an example, incubation of multi‐walled carbon nanotubes with zebra fish embryos led to proliferation deficiency and morphological defects.^27^ As for inorganic nanomaterials, poor pharmacokinetic and pharmacodynamic properties as well as genotoxicity upon interaction with DNA are observed limiting their usage in clinical applications.^28^, ^29^ Toxicity of heavy metal quantum dots are well‐proven^30^, ^31^ and despite gold being regarded as the safest among metallic nanoparticles, its dissociation in the body is rather slow^32^, ^33^, ^34^ which is considered a drawback for clinical application.

Dyes are also considered as organic compounds but unlike carbon nanotubes and graphene‐based materials, they are much smaller in size and have more versatile functional groups therefore their elimination and clearance from the body can be less challenging. This makes them interesting candidates to be explored for biomedical applications.

Dougherty et al. were the first to introduce the first generation of PSs by demonstrating the potential of porphyrins and their derivatives as photoreactive agents for cancer therapy.^35^ Since then, researchers started developing novel PSs which are more hydrophilic in nature and absorb light at higher wavelengths because light penetration through tissues is wavelength dependent. Indeed, light with higher wavelength provides more penetration depth, less scattering and autofluorescence and less photon energy attenuation.^36^, ^37^ As a result, throughout the 1980s, chlorines and cyanines and some of their derivatives were introduced as the second generation of PSs which had improved absorption in the red region.^38^ Nowadays, third generation of PSs consisting of NIR dyes such as indocyanine green (ICG) are employed which have absorption coefficients in the NIR region and benefit from nanotechnological strategies into their design.^39^ In this review, a comprehensive story of the development of different classes of third generation PSs in the context of in vivo applications is illustrated.

### 3.2 Photosensitization: energy dissipation pathways and mechanisms

**FIGURE 1:**
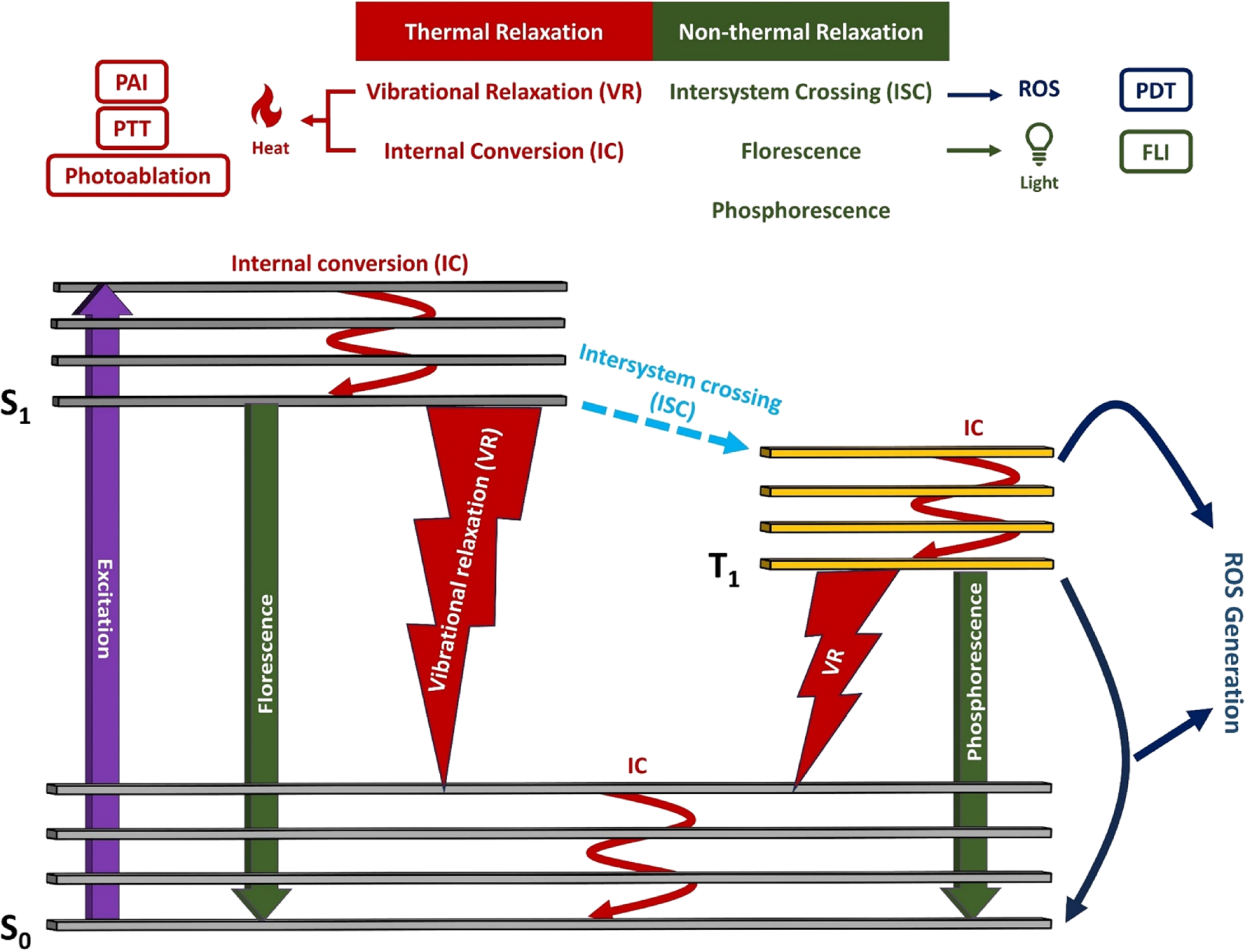
Jablonski diagram and the pathways in which excited PSs go through to reach their previous relaxed state.

The energy dissipation pattern of PSs is explained by the Jablonski diagram (Figure 1). According to this diagram, upon laser light exposure of PSs, excitation, and energy elevation of the electrons from ground state (S_0_) to the singlet excited state (S_*n*_) occurs. Thereafter, rapid energy loss will occur through internal conversion (IC) resulting in thermal energy dissipation from these excited electrons and their subsequent relaxation to the lowest excited singlet state (S_1_). From there, we have categorized energy dissipation patterns into two main pathways to reach S_0_: (a) thermal energy dissipation occurring through vibration relaxation which is non‐radiative in nature and involves the oscillation of the electrons within the PS molecules which can produce heat. This generated thermal energy can be harnessed for photoacoustic imaging (PAI), photothermal therapy (PTT) and photoablation of barriers or biological aggregates. (b) Non‐thermal relaxation which is achieved through two pathways: fluorescence being radiative and including emission of energy in the form of photons with wavelength higher than the incident light which can be employed for fluorescence imaging (FLI) and/or transition from the S_1_ to the triplet excited state (T_1_) and then loss of energy by transferring the excess energy to abundant substrates and/or O_2_ and generating reactive oxygen species (ROS) which can be exploited for photodynamic therapy (PDT).^40^

### 3.3 Dye supramolecular nanoassemblies: towards optimizing photophysical properties

Aside from the typical benefits such as superior tissue distribution and attenuated toxicity provided by nanoparticles, these types of platforms have shown to influence optical and photophysical properties of dyes in positive and/or negative ways.

**FIGURE 2:**
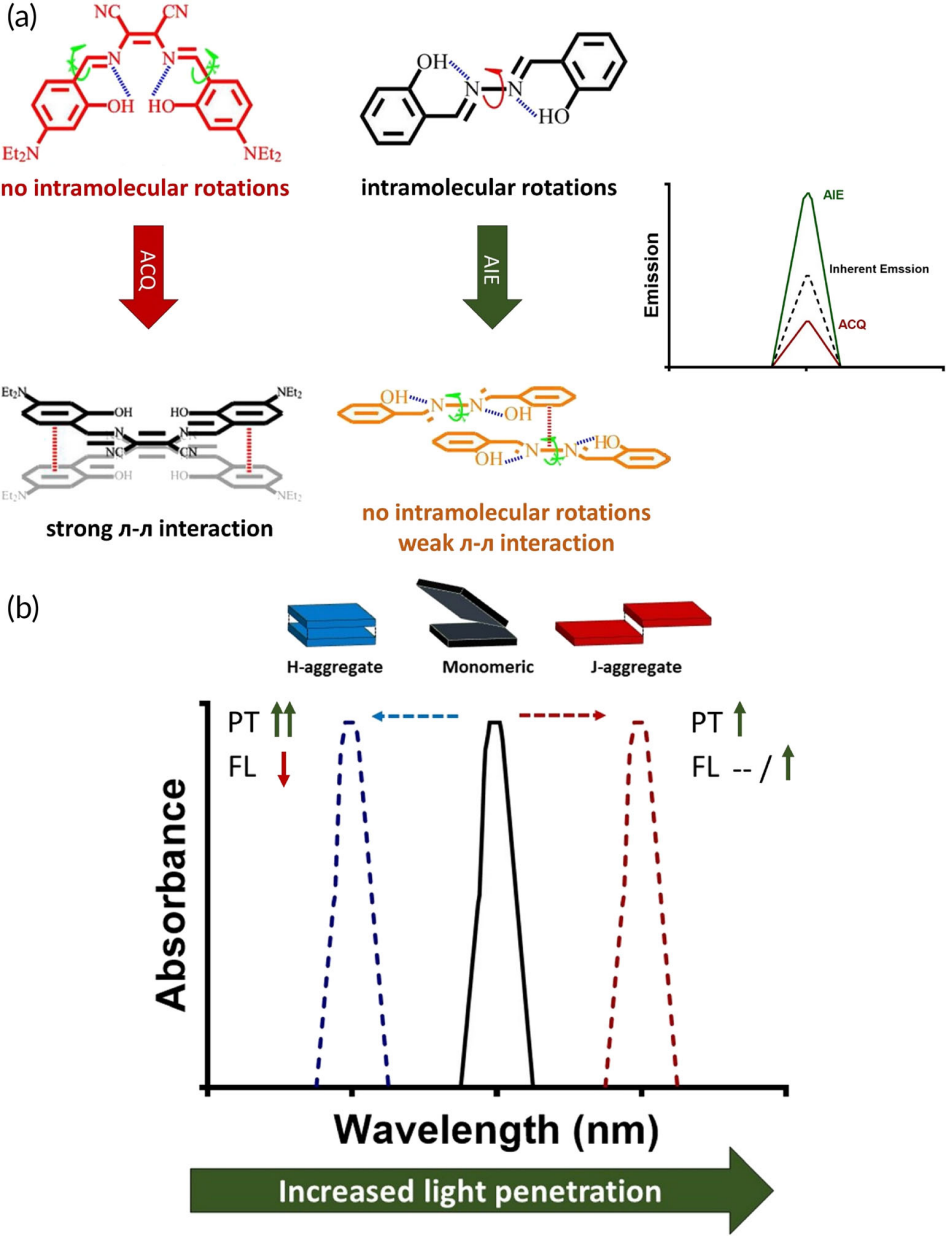
Supramolecular assemblies of dyes impact their photophysical properties. (a) Aggregation caused quenching (ACQ) and aggregation induced emission (AIE) are two phenomenon that oppose one another. In ACQ, emission properties are hampered while AIE luminogens show elevated emission. Adapted with permission from Ref.^41^ (b) Designing nanoparticles and directing their formation towards J and H‐aggregation brings in certain benefits and disadvantages. H‐aggregates exhibit superior photothermal effects with inhibited fluorescence, while J‐aggregates exhibit improved fluorescence and enhanced photothermal properties, but to a lesser extent than H‐aggregates.

Dye molecules can be encapsulated inside NPs using lipid or polymeric adjuvants or form supramolecular self‐assemblies on their own. In some cases, the molecular distribution of dye molecules in these assemblies is as such that tightly packed clusters called aggregates are formed. One of the most frequently‐observed phenomenon associated with aggregation is aggregation caused quenching (ACQ) in which dye molecules are stacked together stochastically through strong л–л interactions^41^ leading to quenched emission and inhibition of ROS generation.^42^, ^43^ While this suppressed emission can be perceived as a negative outcome when for instance, FLI is intended, it can lead to the shift of PS energy dissipation towards the photothermal pathway endowing it superior photothermal properties. On the contrary, some dyes show aggregation induced emission (AIE) upon nanoparticle formation. This phenomenon is usually observed with non‐emissive molecules when they are aggregated in very short proximities to each other which can then regulate their molecular motion (Figure 2a). The underlying mechanism for AIE is described as a restriction in the intramolecular rotation (RIR) and vibration (RIV) which further inhibits the thermal relaxation pathway^44^, ^45^, ^46^, ^47^ resulting in amplified photodynamic and fluorescence (FL) properties.^48^, ^49^

Interestingly, the molecular positioning of some dye molecules can also influence their photophysical properties. There are distinct molecular features that can drive these molecules towards forming orderly aggregation. J‐aggregates are one of the kind in which PS molecules are positioned in a head‐to‐tail conformation in their aggregated state. This conformation can then cause red shift in absorption band and improved absorption coefficient values and, in some cases, can also preserve or even improve fluorescence quantum yield due to improved electronic transitions between PS molecules.^50^, ^51^, ^52^ H‐aggregates are another type of structurally ordered aggregates with the dye molecules positioned parallel to one another. This conformation leads to an overlap of π orbitals and subsequent spectral blue shift and FL suppression followed by higher thermal energy generation^50^, ^52^, ^53^ (Figure 2b). While the negative aspects to this form of aggregation seems off‐putting, their superior photothermal abilities in comparison to J‐aggregates have attracted attention which we will mention further in this review.

## 4 BIOMEDICAL APPLICATIONS OF DYES

The general idea of utilizing dyes as PSs is interesting, both for imaging and therapy in vivo. Yet, application of these molecules for biomedical sake requires optimization to increase imaging and therapy yield and decrease off target effects leading to side effects.

Nanoparticle‐based formulations have been extensively studied in recent years as scientists have grown interest in their potential for various biomedical applications, such as the therapy and diagnosis of diseases such as cancer.^54^, ^55^ Formulating dyes into NPs aims to enhance their accumulation in the target tissue and reduce off‐target body distribution either by passive or/and active targeting.^56^, ^57^ Furthermore, NP formulations can provide stability for dyes that are sensitive to photodegradation. This is pivotal to cyanine dyes of NIR‐I and NIR‐II regions.^58^, ^59^ In this section we will point out to systems in which the building blocks used for fabrication of these NPs are derived either from lipids or polymeric materials that are biocompatible and biodegradable or the NPs are obtained without any adjuvants and through the intrinsic self‐assembly of dye molecules.^60^ In addition, the in vivo applications of these modalities using dye‐based PSs in the form of nanoparticles will be introduced. Finally, a rather unique use of dyes, independent of their photophysical properties, is described. In this application, these molecules are shown to stabilize hydrophobic molecules that have high propensity to aggregation. By employing these dyes, a stable formulation with a high cargo loading can be achieved.

**FIGURE 3:**
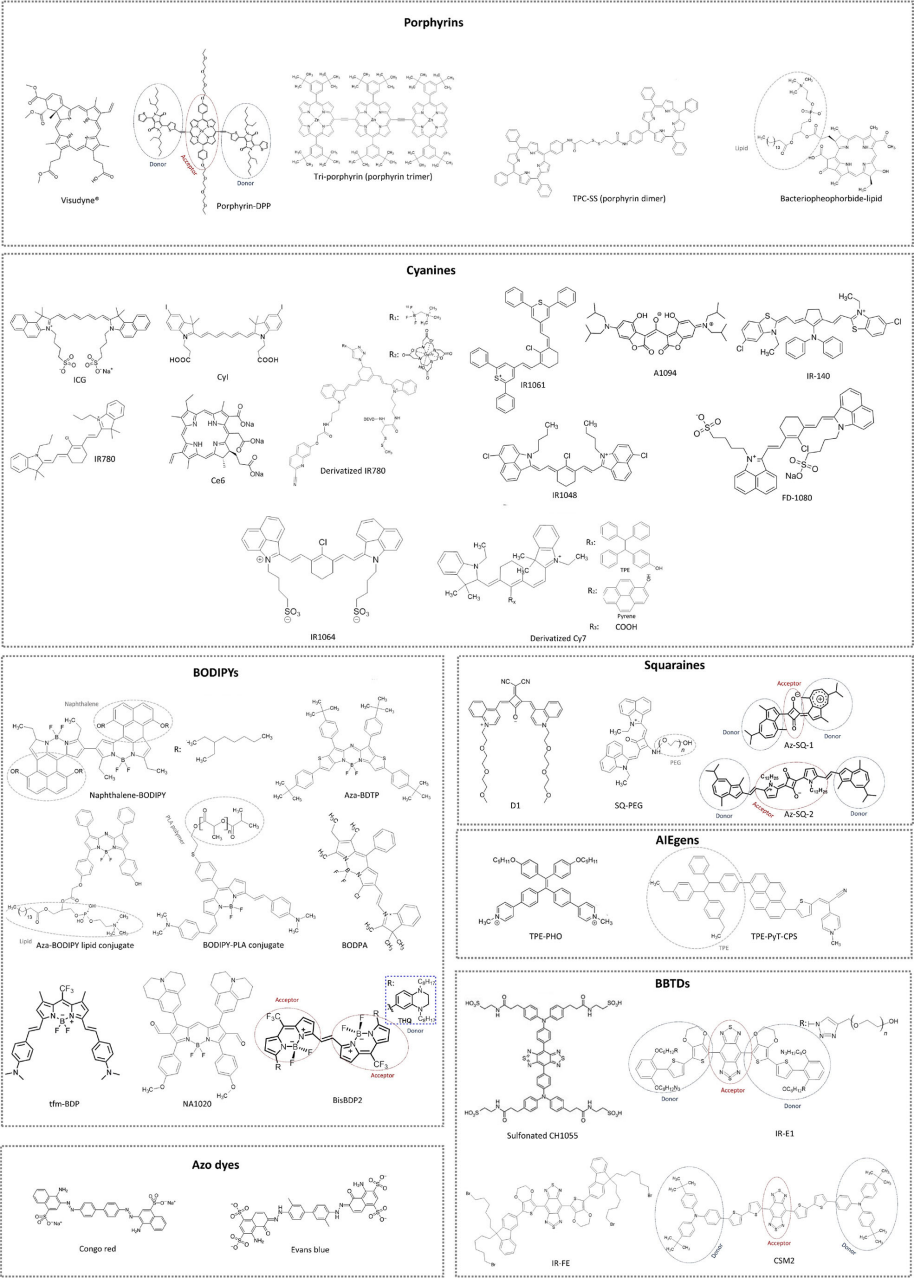
Chemical structures of most discussed dyes.

For a smoother comprehension, the majority of the referred compounds in this paper are categorized in Table 1 along with their chemical structure in Figure 3.

**TABLE 1:** Supramolecular assembly of dyes, their classification, modifications and applications.

Reference	Class	Name	Modification	Modification type	Major NP component	Excitation range	Modalities
87	Porphyrins	Pyropheophorbide‐lipid	Aggregation	ACQ	Self‐assembly	Vis	PAI
88		Bacteriopheophorbide‐lipid		J‐agg	Phosphatidylcholine with varying *T*_m_	NIR‐I	
89		Chlorin dimers (TPC‐SS)	Chemical modification	Dimerization	Self‐assembly	Vis	PAI/PTT/PDT
90		COF‐366 (polymerized porphyrins)		Covalent organic framework	Self‐assembly		
84		Tri‐porphyrin		Trimerization	L‐histidine‐PEG	NIR‐I	PAI/PTT/Chemo
85		Porphyrin‐DPP		D‐A engineering	Self‐assembly		PAI/PTT
152		Octaphyrin		Polymerization	DSPE‐PEG	NIR‐II	PAI
155		BFF	Aggregation	Absorption broadening to NIR‐II	Pluronic F‐127	NIR‐I and NIR‐II	PAI/PTT
187		Verteporfin (Visudyne®)	—	—	DMPC and egg PG	Vis	PDT
189		Ce6	Aggregation	ACQ	BSA‐GO	Vis	PDT
190					Tannic acid‐Fe(III)		
186		Cyl	Chemical modification	Heavy atom effect (iodine)	HA‐PEG‐Cyl self‐assembly	NIR‐I	PDT/PTT
95	Cyanines	ICG	—	—	Mesoporous silica	NIR‐I	PAI
100			Aggregation	J‐agg	DPPC		
97					PLGA‐PEG		
96				—	DOTAP		PTT
176			—	—	DPPC or DOTAP		Photoablation
110		IR780	Chemical modification	^18^F + Casp‐3 substrate grafting	In situ self‐aggregation upon degradation by Casp‐3 in the presence of GSH		PAI/PET
111				Gd + Casp‐3 substrate grafting			PAI/MRI
112			—	Electrostatic conjugation	Tetrahedral DNA (Td)		FLI/PDT/PTT
226		IR783	—	—	Self‐assembly		Stabilizer
113		IR820	—	—	Human cell membrane		FLI/PAI/PTT
115		Cy7	Chemical modification + aggregation	Pyrene, TPE and COOH addition + H‐agg	Self‐assembly		FLI/PTT
117		QCy		Quinoline addition + H‐agg		NIR‐I	PAI/PTT
142	Cyanines	A1094	Aggregation	—	RGD‐HepB core protein	NIR‐II	PAI
143				J‐agg	DSPE‐PEG		
145		IR1048	—	—	DPPC/4T1 cell fragment coated		PAI/PTT
146		IR1064	—	Gd (metal) incorporation	Self‐assembly modulated with Gd		MRI/FLI/PAI/PTT
147		IR1061	—	—	DSPC		PAI/PTT
118			Aggregation	H‐Agg	DPPG	NIR‐I and NIR‐II	FLI/PTT/Chemo
217		IR‐140	Aggregation	J‐Agg	Mesoporous silica	NIR‐I	FLI
218		FD‐1080			DMPC	NIR‐II	
219					Heating dye in H‐Agg form		
122	BODIPYs	Naphthalene BODIPY (Na‐BD)	Chemical modification	Dimerization + naphthalene fusion	BSA	NIR‐I	PAI
123		Aza‐BDTP	Aggregation	ACQ	Plutonic F‐127		PAI/Chemo
124		Aza‐BODIPY‐lipid	Aggregation	J‐agg	DPPC		PAI/FLI
125		BODIPY‐PLA	—	—	PEG‐PLA		PAI
126		Semi‐cyanine‐BODIPY (BODPA)	Chemical modification	H_2_S sensing site (CI atom)	Silica nanocomposites		PAI
70		CF_3_‐BODIPY (tfm‐BDP)	Chemical modification	Substitution of H or CH_3_ with CF_3_	DSPE‐PEG		PAI/PTT
156		NA1020	Chemical modification	Modification of the acceptor unit + NO donor encapsulation	Poloxamer	NIR‐II	FLI/PTT/Gas
157		BisBDP2	Chemical modification + aggregation	D‐A‐π‐A‐D structure + J‐agg	Plutonic F‐127		PAI/PTT
136	Squaraines (SQs)	SQ‐PEG	Aggregation	H‐agg (H dimers)	Self‐assembly	NIR‐I	PAI/PTT
137		Azulene‐SQs (Az‐SQ1 and 2)	Chemical modification + aggregation	D‐A‐D engineering + J or H‐agg	DSPE‐PEG		PAI/PTT
135		D1	—	—	Pluronic F‐127		FLI/PAI
191		SQ‐peptide	Chemical modification + aggregation	Cathepsin‐B reactive site + ACQ	Iron oxide	Vis	MRI/FLI/PDT
193	AIEgens	TPE‐PHO	Aggregation	ACQ + AIE	Calixarene	Vis	FLI/PDT
196		TPE‐PyT‐CPS		AIE	Self‐assembly		PDT
209	BBTDs	Sulfonated CH1055 (CH‐4T)	Chemical modification	Sulfonation for protein conjugation	Albumin	NIR‐I	FLI
210		IR‐E1		D‐A‐D engineering	Self‐assembly		
214		IR‐FE	—	—	Polystyrene‐DNA		
153		CSMN2	Chemical modification	D‐A‐D engineering	Pluronic F‐127	NIR‐II	PAI/PTT
154		IR‐SS		D‐л‐A‐л‐D engineering	DSPE‐PEG		
224	Azo dyes	Evans blue (EB)	—	—	Self‐assembly	Vis	Stabilizer
224		Congo red (CR)					

### 4.1 Photothermal applications

Photothermal applications exploit PS potential to generate heat when exposed to light. The dissipated heat from the PS can then be either used for photothermal therapy (PTT) of cancer through mediating cellular death mechanisms (i.e., apoptosis and necrosis)^61^, ^62^ or cancer diagnosis by generating photoacoustic signals as a result of the sudden temperature rise of the media surrounding the PS which then provides the possibility of photoacoustic imaging (PAI).^63^ Optimal PTT and PAI are achievable with dyes that own high molar absorption coefficients as well as higher tendency towards undergoing non‐radiative decay.^64^

Recently, researchers have grown interest in another photothermal phenomenon namely the generation of vapor nanobubbles (VNBs) which emerge from PSs upon irradiation with a pulsed laser (i.e., nanosecond, picosecond or femtosecond lasers). The irradiated pulsed light can lead to a rapid and enormous increase of PS temperature leading to the evaporation of the surrounding water rapidly causing the formation of VNBs. Upon consumption of the energy by the surrounding water, the VNB collapses which imposes sheer mechanical forces to biological matter such as cell membrane or biological barriers^65^ which will be discussed further.

#### 4.1.1 Dyes used for PAI and PTT

A wide variety of dye molecules are now available for PAI and PTT. A first major specification which makes these molecules different from one another is their ability to absorb light closer to the IR region since higher wavelengths provide higher tissue penetration and a better image contrast when PAI is performed.^36^, ^66^ This improvement is attained from minimized scattering and light absorption by unwanted components.^67^, ^68^ A second major specification is the possibility to improve the photothermal conversion efficacy (PCE) of these molecules which facilitates imaging and therapeutic performance and is crucial to obtain high function yield.^69^, ^70^ This calculated PCE value is often expressed in percentage and can vary based on the type of PS. For example, plasmonic nanomaterial PCE is explained by the Mie‐Gans theory which demonstrates that smaller size, larger aspect ratio and higher absorption efficiency are determinant factors for optimal PCE values.^71^ While many of these materials have exhibited PCEs above 90%, there are no clear standards to rank PSs based on their PCE as gold nanorods and gold nanoshells with values of 21% and 13% are commercialized.^72^ As another example, a value of 40% was reported for polydopamine (PDA) NPs which are classified as semiconducting polymers.^73^ PCE of dyes, on the other hand, very much depends on their chemical structure and supramolecular conformation when formed into nanostructures. More details will be clarified further in this review.

Melanin can be exemplified as an endogenous dye that has previously been used in NP form for PAI and PTT.^73^, ^74^, ^75^, ^76^, ^77^ The success with melanin PSs has inspired researchers to investigate PDA NPs which are formed synthetically from dopamine monomers.^78^, ^79^, ^80^ These NPs are not a subject of this review as they are classified as semiconducting polymers. Porphyrins are other endogenous molecules^81^, ^82^ that contribute to hemoglobin structure as heme cofactor and play a role in the activity of redox enzymes such as cytochromes.^83^ Since most porphyrins have an absorbance below 700 nm^84^ and undergo radiative decay in the form of fluorescence resulting in low photothermal conversion efficacy (PCE),^85^, ^86^ efforts were made to increase their absorbance towards NIR and enhance PCE by formulating them in NPs. Lovell et al.^87^ improved porphyrin PCE by synthesizing a phospholipid‐dye conjugate and fabricating NPs from it. They were able to amplify PA signal intensity and impart photothermal therapy properties in vivo due to self‐quenching of porphyrins in the bilayer. This quenching method is intended to hamper PS radiative decay and thus shift the balance towards thermal energy dissipation. In a later study, based on the same hypothesis, the same group managed to fabricate a thermosensitive photoacoustic nanoswitch. This time, they sought to improve the photophysical properties of PSs by forming J‐aggregates inside the vesicles. In their research, liposomes consisting of lipid–porphyrin conjugates together with other lipids were formulated. It was shown that J‐aggregation led to a red shift in optical density along with 2.7‐fold absorptivity enhancement. They were also able to evaluate temperature changes in vivo because of the disorganization of J‐aggregates in liposomes upon a certain temperature variation.^88^ As an alternative strategy, altering the porphyrin molecule itself was explored. Modifications such as inclusion of quinone along with different transition metals,^86^ conjugation of electron donors,^85^ synthesis in dimerized^89^ and covalent organic frameworks comprising of multiple porphyrin molecules attached in a network structure^90^ have been reported previously. Most recently, Zhang et al.^84^ formulated porphyrin trimers into NPs via supramolecular conjugation to histidine‐modified PEG. To enhance therapeutic efficacy, they also co‐loaded doxorubicin into their NPs. Upon irradiation of the tumor with 808 nm laser for 5 min, temperature elevation up to 47°C was observed which then led to tumor growth suppression. Further evaluation of cell death mechanism revealed apoptosis to be responsible for ~50% of the cell population.

**FIGURE 4:**
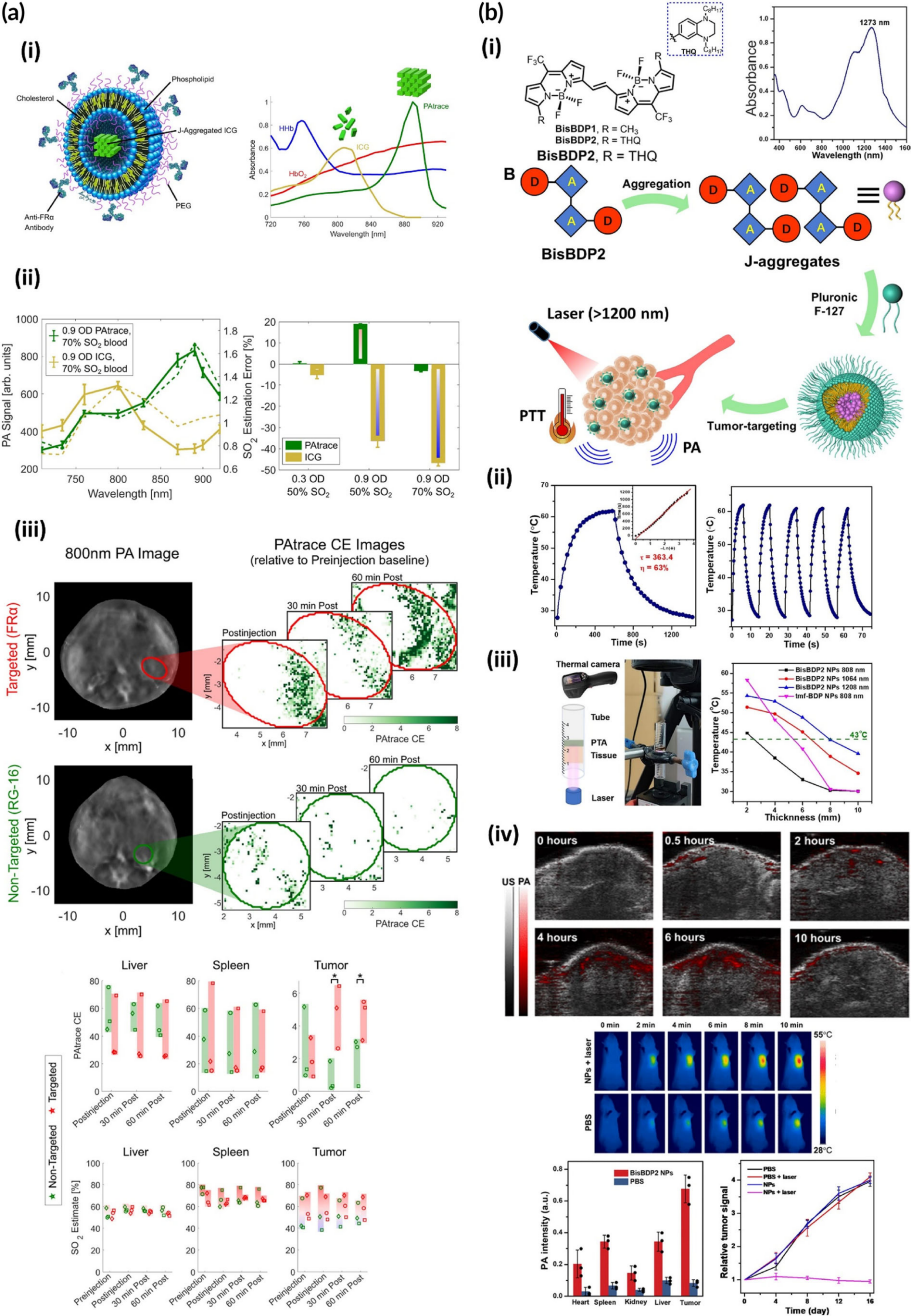
Dye supramolecular nanoassemblies for in vivo PAI and PAI‐guided PTT. (a) (i) Formation of J‐aggregated ICG liposomes (PAtrace) and their unmixed optical absorbance spectra when compared to other blood elements and monomeric ICG in blood serum. (ii) Spectral data from multi‐wavelength PA acquisitions (left graph) are shown by solid lines (error bars indicate mean ± SD across 12 *z*‐slices), while theoretical spectra for these combinations are indicated by dashed lines. The right graph shows that the PA‐based SO_2_ estimation error for all concentration/SO_2_ combinations tested is less than 20% absolute error for PAtrace (green bars), while it is nearly 50% error in the presence of ICG (gold bars). (iii) In vivo PAI and SO_2_ evaluation for targeted FRα‐PAtrace (red) and non‐targeted RG‐16‐PAtrace (green): Representative 800 nm PA image of SKOV3 tumors with ROIs indicated by red/green ellipses. Representative PAtrace contrast enhancement (CE) images in post‐injection time‐points of targeted FRα‐PAtrace and non‐targeted RG‐16‐PAtrace. PAtrace CE in the liver (left) and spleen (middle) ROIs suggest no significant difference between FRα‐PAtrace and RG‐16‐PAtrace liver or spleen accumulation whereas PAtrace CE in tumor ROIs (right) presents positive enhancement for both targeted and non‐targeted PAtrace immediately post‐injection, but with targeted enhancement significantly greater 30 min (*p* = 0.017) and 60 min (*p* = 0.044) post‐injection. PA‐based SO_2_ estimates for liver (left), spleen (middle), tumor (right) ROIs show that SO_2_ does not change significantly through time, indicating no substantial physiological changes occurred during imaging. Adapted with permission from Ref.^100^ (b) (i) BisBDP2 J‐aggregates stabilized with pluronic F‐127 for PA guided PTT with abs. maxima at 1273 nm. (ii) The NPs with PCE calculated to be 63% and temperature reaching above 60°C with five duty cycles. (iii) A schematic of the apparatus used for in vitro evaluation of PTT (left) and comparison of temperature increase as function of treatment depth for BisBDP2 NPs with NIR‐I PT agent with highest reported PCE (right) showing that with NIR‐II NPs, even at 8 mm, treatment temperature of 43°C can be reached. (iv) In vivo evaluations: in vivo PA images of orthotopic liver tumor using 1260 nm laser (top) and tumor temperature increase observed by infrared thermal images. Bottom row showing highest PA intensity in tumor site (left graph) and successful tumor growth suppression after treatment (right graph). Adapted with permission from Ref.^157^

In recent years, researchers have shown interest in developing exogenous PS that intrinsically exhibit NIR properties. For this purpose, cyanine dyes have been studied extensively since these molecules show high NIR molar extinction coefficient and fluorescence quantum yield which are crucial to obtain high brightness and contrast for fluorescence imaging.^91^ However, for photothermal applications, improving the PCE to achieve high thermal energy dissipation is of prime importance. On the other hand, some of these molecules like indocyanine green (ICG) which is FDA‐approved for imaging and is one of the most widely used dyes for biomedical applications, have poor solubility and photostability,^92^, ^93^ and are prone to aggregation.^94^ These limitations hamper its biomedical application hence its formulation into NPs has been investigated. To overcome these limitations and enhance photothermal properties, Chaudhary and coworkers evaluated the effect of loading ICG into physical (lipid‐bilayer‐coated (LB) and layer‐by‐layer polyelectrolyte‐coated (LBL)) and chemical (covalently‐bond NH_2_ and PO_3_ groups) surface functionalized mesoporous silica NPs (MSNs). All MSNs provided complete stability of the dye compared to 20% degradation of the dye in its free form after 3 h. Most importantly, the highest PA intensities were achieved with NH_2_‐MSNs and LBL‐MSNs which exhibited around 1.4‐fold stronger PA signal compared to ICG monomers in vivo.^95^ This can be attributed to the positive charge of NH_2_ and chitosan (first layer of coating in LBL‐MSNs) which induces aggregation of the negatively charged ICG and a better preservation inside the NPs. This is also in‐line with results from Lovell group showing improvement in PA intensity upon ICG aggregation to positively charged DOTAP liposomes.^96^ Considering J‐aggregate potential for improving optical properties of many dyes like ICG, researchers have focused on directing aggregation towards J‐aggregates. Changalvaie and coworkers encapsulated small ICG J‐aggregates into PEG‐PLGA polymersomes to achieve PAI successfully^97^ nevertheless, the formulation process described in their paper seems difficult to execute for high throughput production. Alternatively, using delivery platforms as surfaces for in‐situ formation and hosting of J‐aggregate appears an easier strategy for execution. In this approach, these aggregates are stabilized by the assistance of lipid bilayers.^98^, ^99^ In this case, higher J‐aggregate formation yield is shown to be the outcome when using lipids with higher phase‐transition temperatures or lower bilayer fluidity. In 2021, Wood et al.^100^ fabricated folate receptor alpha (FRα) decorated rigid ICG J‐aggregate liposomes mainly composed of DPPC which they named PAtrace (Figure 4ai). One of the benefits of employing J‐aggregated ICG over the monomeric form was to avoid spectral mixing between hemoglobin and the PS. This spectral unmixing would then enable dynamic tumor microenvironment evaluation by oxygen saturation (SO_2_) measurement in the presence of the PS which is important for evaluating tumor stage.^101^ PAtrace was shown to absorb between 870 and 920 nm. In this wavelength range, the absorption of hemoglobin is relatively flat whereas monomeric ICG absorption interferes with the one of hemoglobin (Figure 4ai). Therefore, spectral unmixing feasibility was proven in vitro by measuring oxygen saturation (SO_2_) accuracy using PAtrace and monomeric ICG in a tissue‐mimicking (TM) phantom using seven different wavelengths starting from 710 to 920 nm. Comparison between the calculated theoretical absorption spectra of ICG and PAtrace with their acquired PA signals in the presence of 70% SO_2_ blood clearly indicates divergence for ICG monomers with correction coefficient of 0.44 in contrast to 0.96 for PAtrace showing a well matching pattern. The estimated SO_2_ values only exhibited a maximum of 18% error for PAtrace as opposed to ICG with a calculation error of >30% (Figure 4aii). Further in vivo experiments validated PAI contrast enhancement by PAtrace even without the FRα targeting moiety though this moiety provided a much more significant contrast enhancement compared to the non‐targeted form. SO_2_ estimations in three different organs including the tumor were also performed at separate timepoints post‐injection in vivo in which tumor, liver and spleen oxygen levels were observed to be constant (Figure 4aiii). Besides PAI, improved photothermal properties of ICG in J‐aggregated form has led to systems for employment in PTT alone or in combination with other modalities.^102^, ^103^

Regardless of the fact that FDA‐approved ICG has attracted a lot of interest in recent years, its instability and rapid in vivo degradation in aqueous solutions^104^ has driven researchers to explore other molecules of the cyanine family. IR780 has been a promising alternative because of the rigid cyclohexane in the heptamethine chain providing more stability^105^ and enhanced fluorescence intensity by reduced quenching^106^ enabling bimodal PAI and FLI along with PTT.^107^, ^108^, ^109^ Furthermore, because of the reactive Cl‐containing cyclohexane ring, more versatility is provided for conjugation to other compounds. Using this moiety, Ye's group conjugated this dye to radioactive ^18^F and Gd to endow PET^110^ and MRI^111^ in addition to PAI respectively. These conjugates were tailored to be reactive to caspase‐3 overexpressed in tumor. Upon in‐situ accumulation of the conjugate, the caspase‐3 reactive site is cleaved enabling the formation of macrocycles which then would lead to in‐situ formed NPs with enhanced PA, PET and MRI signals. In contrast to chemical conjugation, Wang et al.^112^ conjugated positively charged IR780 to the negatively charged tetrahedral DNA (Td) through electrostatic interactions and л–л stacking. This conjugation resulted in the formation of NPs that could deliver the dye to the tumor only through the EPR effect. These NPs showed superior PTT capabilities which was concluded from improved PCE in vitro. The in vitro PCE was evaluated based on measuring FL intensity of MCF‐7 cells treated with IR780 and IR780@Td NPs and comparing these intensities before and after laser treatment. It was observed that FL intensity of the NP treated cell group decreased in a more significant manner after laser treatment compared to IR780 treated cells. This higher fall of FL signal was ascribed to the higher reduction of radiative decay that can shift the balance towards improved photothermal effects. In vivo PTT evaluation by measuring infrared tumor images and tumor temperature measurements during treatment in addition to tumor volumes after treatment also confirmed the advantage of using IR780@Td NPs over IR780. Other cyanine dyes such as IR820,^113^ IR825^114^ have also been investigated for the same purposes where multimodal functionalities were the main objective and because they had red‐shifted absorptions. Seemingly, application of these cyanine molecules can be of benefit for imaging PAI and FLI along with PTT in the NIR‐I window.

As explained before, J‐aggregates have excellent biomedical properties, especially when multimodal therapeutic application is intended. However, their molecular alignment does not inhibit the radiative channel which can retard the photothermal properties. Therefore, H‐aggregates are a better alternative as their formation elevates these properties through impeding fluorescence providing superior photothermal properties compared to J‐aggregates. Works of Wu and Feng in which Cy7 was molecularly engineered with conjugation of pyrene, tetraphenylethene (TPE) or two carboxylic acid groups together with copper, clearly highlight the photothermal potential of these nanoaggregates however the PCE was rather low (~22%).^115^, ^116^ Later, Wei et al.^117^ designed a quinoline incorporated cyanine dye (QCy) owing a PCE of ~60% with the aim of modulating the planarity of the molecule to be stationed in a face‐to‐face position. Tumor growth was inhibited twice as much, compared to the non‐H‐aggregated form after PTT in vivo. However, the absorption shift of the NIR dye towards the visible region was a clear indication of the drawback of using these aggregates. In a similar fashion to J‐aggregates, some dyes can form H‐aggregates without the need for any chemical modification and only through the addition of an appropriate NP forming component. Yu's work is a good example in which H‐aggregates of the readily available NIR‐II absorbing dye IR1061 were formed in the negatively charged DPPG lipid bilayer of liposomes.^118^ In their work, the extent of aggregation was calculated by molecular dynamic simulation in order to achieve optimal H‐aggregation. Interestingly, since not all dye molecules participate in the structure of the H‐aggregate and a portion of the molecules are located in the bilayer in their monomeric form, NIR‐II FLI was performed using 1064 nm laser benefiting from dye monomers in addition to PTT with 808 nm laser wavelength benefiting from dye H‐aggregates. This study clearly brings this idea into prospect that by adjusting the H‐aggregate to dye monomer ratio, both thermal and non‐thermal properties could be exploited.

BODIPYs are a well‐known class of dyes known to exist since 1968.^119^ They possess high photostability and straight‐forward synthesis^120^ but tend to undergo radiative decay which is a non‐photothermal process. Chemical modification of these dyes have shown to be effective in promoting non‐radiative decay and shifting the absorption spectra towards NIR region.^121^ For example, Wu and coworkers studied PA activity of naphthalene‐fused BODIPY derivative (Na‐BD) compared to ICG and concluded better PA signal and stability. Next, the dye was aggregated on bovine serum albumin (BSA) and successfully employed for in vivo PAI in a liver cancer mouse model.^122^ Later on, Zhang and co‐workers prepared a novel micellar formulation of an aza‐BODIPY derivative, namely β‐thiophene‐fused BF_2_‐azadipyrromethene (aza‐BDTP), a hydrophobic and NIR absorber similar to Na‐BD, in combination with paclitaxel (PTX) for combinatorial PAI/chemotherapy. Both hydrophobic aza‐BDTP and PTX were encapsulated into pluronic F127 micelles by employing the simple surfactant‐stripped induced frozen micelle preparation method in which pluronic F127 micelles were formed under 4°C (the critical micellar temperature (CMT)) and thereafter filtered to remove the loosely‐bond and free pluronic F127 resulting in pure micelle formulation. The formulation demonstrated enhanced PA capacity in vivo due to improved PCE. This happened because of aggregation‐caused quenching (ACQ) of aza‐BDTP upon accumulation in the core of the NPs which shifted the energy dissipation pattern off the PS towards thermal decay.^123^ Another example of aza‐BODIPY formulations is a liposomal formulation of aza‐BODIPY in the form of lipid conjugate and in variable percentage of the conjugates in order to form J‐aggregates in NPs. This was done with the intention of improving optical properties of the dye with the methodology inspired from their previous success with the work on porphysomes.^87^ The acquired so‐called BODIPYsomes had absorption maxima shifted towards the NIR‐I. Increasing the percentage of BODIPY–lipid conjugate resulted in improved fluorescence quenching therefore higher PCE. In vivo PAI resulted in optimal imaging at around 700 nm while evaluating mice bearing prostate cancer tumor.^124^ Alternatively, BODIPY‐polylactic acid (PLA) conjugates were also fabricated by synthesizing PLA using ring‐opening polymerization on the BODIPY template and incorporated into polymersomes along with PEG‐PLA. Successful employment of this system for in vivo PAI further into the NIRI window was demonstrated in the kidney of healthy mice.^125^ Not only can BODIPYs be used for cancer PAI, but they offer a promising platform for quantification and analysis of different elements due to their versatility in functionalization enabling them to be tailored thus activated in the presence of a specific ligand in vivo. Previously, endogenous H_2_S tracking by PAI was reported using semi‐cyanine‐BODIPY loaded NPs in HCT116 tumor‐bearing mice in vivo.^126^ However, the application of these dyes for non‐cancer related PAI such as ratiometric detection of copper,^127^ fluoride^128^ and even H_2_S levels in liver injuries^129^ is performed in free form since site‐specific accumulation is not required and systemic measurement is desired. The highest reported PCE belongs to CF_3_ attached BODIPY namely tfm‐BDP. This construct has an absorption peak of ~810 nm and a PCE of 88%. The CF_3_ moiety is deemed to be responsible for this exceptional PCE through its barrier free rotation meaning the group is not constrained to any energy barriers thus it can rotate freely to dissipate energy which then ensures high thermal energy generation. Consequently in vivo PAI guided PTT on 4T1 tumors using 808 nm laser resulted in successful image‐guided photothermal therapy.^70^ Even though BODIPYs have been commonly tailored and formulated, only a few of these dyes have been reported to have absorbance above 800 nm^130^ which can be considered a drawback compared to other probes.

Squaraines (SQs) are a class of dyes extensively used in FLI due to their radiative decay characteristics.^131^ The first reported photothermal‐based applications of these dyes dates back to 2014 where SQs were aggregated to BSA forming BSA‐nanocomplexes for in vivo liver and tumor PAI^132^ and PTT.^133^ Later works intended to benefit from encapsulation of both aggregated and monomeric forms to obtain simultaneous photoacoustic and fluorescence properties of SQs respectively.^134^, ^135^ In their works, they have demonstrated the use of H‐dimers and/or aggregates in balance with the monomeric form of selected SQs to achieve both modalities. The unique photothermal properties of H‐aggregates motivated Wang et al.^136^ to design H‐dimer self‐assemblies of SQs by conjugation to PEG and formation of dye nanospheres. Intriguingly, they were able to reach a PCE higher than 80%. There are examples where only chemical engineering without any intention to induce aggregation was employed to improve the thermal energy dissipation profile. For instance, Yao et al. synthesized high performance photothermal agents consisting of azulene‐containing SQs. By grafting the azulene component to the SQ molecule and in the form of NPs, they achieved faint fluorescence improving non‐radiative decay resulting in PCE up to ~50% and 15‐fold signal to noise ratio (SNR) in one of the probes in vivo. By in vivo tumor treatment using 808 nm laser wavelength, a temperature as high as 65°C was reached only 2 min into the treatment and after 8 days, the tumor was successfully ablated.^137^ Despite efforts made to improve the photothermal properties of these agents, they seem to be less interesting candidates when compared to other dyes, to be employed for PAI and PTT as their absorption is limited to the NIRI and in the majority of cases, PCE reaches around 50% or lower.^138^, ^139^

All the previously mentioned small‐molecule dyes own excitation wavelength in the region of NIR‐I (700–1000 nm) which already provide penetration depth of up to 3.5 mm compared to 1mm for visible light.^140^ However, incident light with wavelength in the NIR‐II (1000–1700 nm) region has shown to boost PAI and PTT depth reaching up to 10mm owing to lower interactions with biological tissues.^141^ In 2019, Li et al. discovered the A1094 molecule for PAI in NIR‐II. The molecule belongs to the croconaine subclass of the cyanine dye family. A 30 nm NP composed of hepatitis B virus core protein decorated with RGD peptide (RGD‐HBc) was designed and loaded with A1094. Upon aggregation‐induced absorption enhancement (AIAE) inside the core of the carrier, PA signal amplification was acquired up to 9‐folds allowing imaging depth of ~6 mm for in vivo glioblastoma imaging with 1064 nm incident light wavelength.^142^ Inspired by this work, the group fabricated 100 nm DSPE‐PEG2000 micelles containing j‐aggregates of A1094.^143^ Other cyanine dyes such as IR1048,^144^, ^145^ IR1061 and IR1064^146^, ^147^ have been utilized in a similar fashion in which the extended л‐conjugation has improved their maximum absorption wavelength compared to the conventional NIR‐I cyanine dyes.^148^ As expected, agents that are applied in PAI have the potential to be used for PTT as well. For instance, Hong et al.^144^ applied IR1048 for PAI guided PTT of bladder orthotopic tumor in mice. In their system, the dye had been loaded into hyaluronic acid (HA)‐coated liposomes in order to actively target bladder tumor cells which are CD44 positive. Results indicated significant PTT efficacy and difference in group treated with the uncoated liposomes highlighting the value that precise choice of targeting can bring. This efficacy evacuation was performed through measuring tumor bioluminescence as well as visual inspection images of the tumor after day 14. PAI data also confirmed tumor destruction during treatment.

Besides cyanines, with the same design rationale, porphyrin dyes were also structurally expanded to reach the NIR‐II region. Derivatives such as metal‐complexed hexaphyrins were developed by Furuta and collaborators, reaching absorbance even in the NIR‐III region, however, their biological application had yet not been evaluated^149^, ^150^, ^151^ thus Sessler et al. developed DSPE‐PEG2000 NPs loaded with octaphyrin which generated PA signals upon exposure to acidic pH and environment with reducing properties as in tumors. They were able to validate PAI with studying healthy stomach and monitoring its performance. They could also image HepG2 tumors in mice in which 42fold discrimination in signal of the cancerous tissue was obtained.^152^ As an alternative to the expansion of л‐conjugation and metallic moiety inclusion, employing donor–acceptor–donor (D‐A‐D) structures in dye molecules is also being explored and benzo[1,2‐c:4,5‐c′]bis([1,2,5]thiadiazole) (BBTD) dyes have been introduced.^153^, ^154^ However, the process of synthesizing NIR‐II absorbing molecules is rather challenging and current available library of molecules is limited, thus Xiang et al. developed an NIR‐II organic absorbing NP by simple aggregation of an NIR‐I absorbing dye namely boron difluoride formazanate (BFF) inside NPs constructed with Pluronic F127. The aggregated dye had a broad absorption spectra due to л–л stacking interactions inside the NPs making it feasible to perform NIR‐II PAI‐guided PTT of hepatocellular carcinoma in vivo.^155^ In their study, two conditions were tested in mice bearing orthotopic tumors; a deep tissue mimicking condition in which 4 mm chicken breast tissue layer was located on top of the tumor site and a condition without tissue layer. When mice received treatment with both NIR‐I and NIR‐II conditions, the normal orthotopic tumor was ablated similarly whereas in the deep tissue model, only the NIR‐II laser could ablate the tumor. This observation underlies the value PSs reacting to NIR‐II bring to treatment compared to NIR‐I PSs.

While currently, most reported NIR‐II organic single small molecules belong to the cyanine family, there have been new reports in which BODIPYs were engineered to perform in the NIR‐II window. For instance, using molecular engineering, a novel NIR‐II aza‐BODIPY molecule, namely NA1020 was designed and used in vivo for PTT of osteosarcoma in combination with a thermal‐sensitive NO donor using poloxamer as their NP forming agent.^156^ In another study, to further optimize properties of BODIPY PSs, chemical engineering was performed to also guide the dye towards forming J‐aggregates. Thus, two identical BODIPY molecules containing tetrahydroquinoxaline (THQ), were fused together by an ethenylene bridge. The D‐A‐π‐A‐D molecular formulation endowed the dye an absorption peak of 1100 nm.^157^ Due to the zig–zag design of the molecule, the dye showed excellent J‐aggregation behavior which shifted the absorption peak to 1300 nm. By stabilizing the J‐aggregates into NPs using pluronic F‐127, PAI guided PTT was executed with irradiation of 1208 nm laser (Figure 4bi). The obtained aggregated dye had PCE of ~60% and maintained stability and performance upon laser irradiation up to five cycles (Figure 4bii). To take further steps towards in vivo PAI‐guided PTT, firstly the PAI ability of the PS was tested by visualizing orthotopic hepatocellular carcinoma tumor on a mouse model using laser above 1200 nm. The PA signal from the PS in the tumor was at its highest 6 h after PS injection and this signal intensity inside the tumor was observed to be two to five times higher than the four other organs providing great image contrast. Thereafter, the photothermal mechanism validation and its outcome were evaluated by (a) obtaining thermal images from the tumor site which showed temperature rise as high as ~50°C in a course of 10 min. This value is above the substantial temperature needed for PTT and (b) monitoring tumor growth which demonstrated growth suppression 2 weeks following the treatment (Figure 4biv). Noteworthy, to highlight the importance of employing NIR‐II systems, tumor treatment depth of their system was compared with a previously reported NIR‐I BODIPY dye with 88% PCE^70^ by temperature measurement in vitro. It was identified that the novel NIR‐II NPs were able to provide sufficient treatment temperature of 43°C with depth of up to 8 mm as opposed to the NIR‐I NP with ~5 mm treatment depth (Figure 4biii).

#### 4.1.2 Photoablation of biological aggregates and barriers using dyes

**FIGURE 5:**
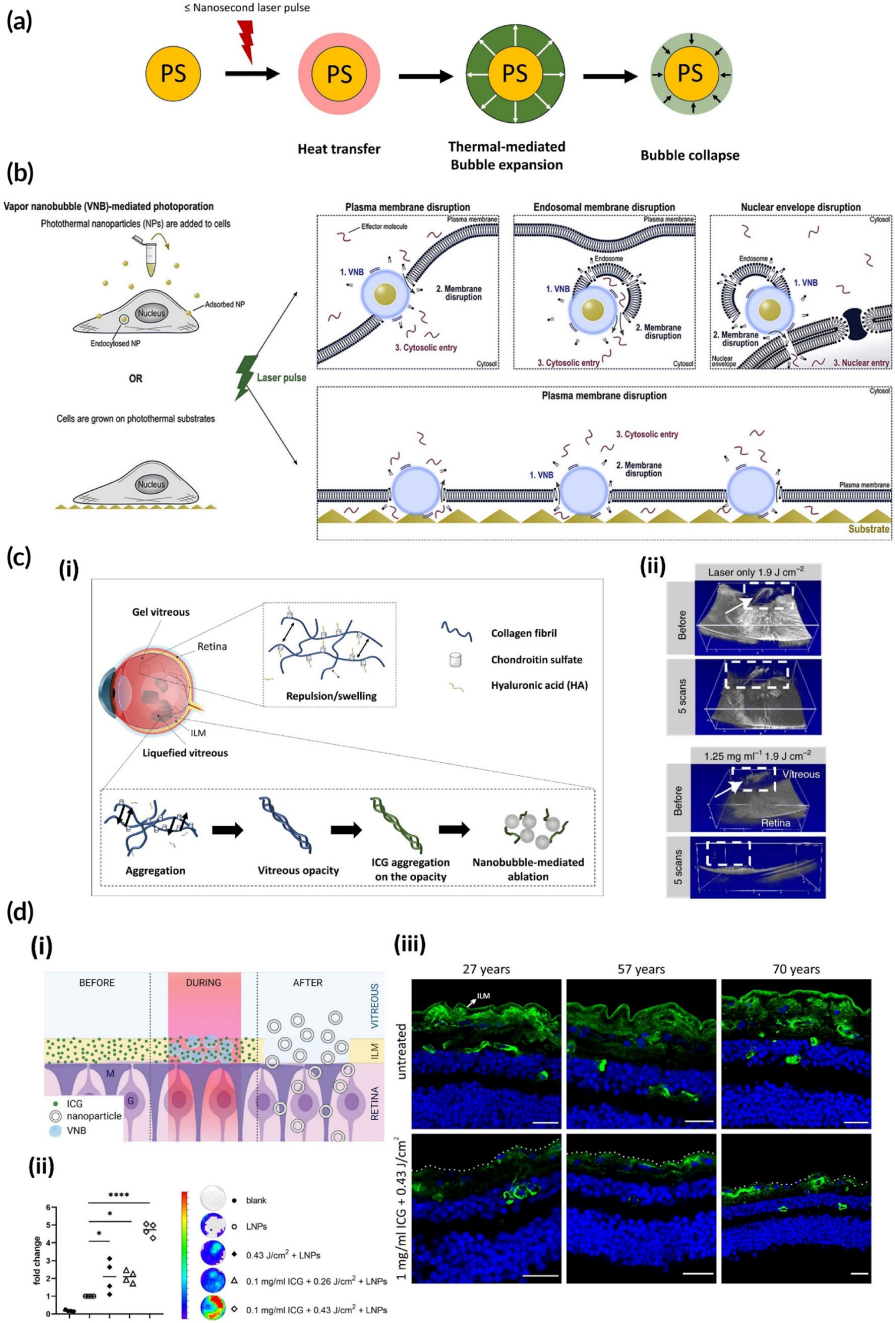
Dyes have the potential to generate VNBs upon irradiation with a pulsed‐laser. (a) The process of the generation of vapor nanobubbles (VNBs). (b) VNBs for photoporation at the level of cell, endosome and nucleus membranes in order to deliver molecules. Adapted with permission from Ref.^65^ (c) (i) Photoablation of vitreous opacities using pulsed laser and ICG. (ii) OCT images of the fibers in rabbits in vivo showing ablation while no destruction was observed after treatment with the laser only. Adapted with permission from Ref.^173^ (d) (i) Photodisruption of the ILM using laser and ICG. (ii) Retinal photoluminescene measurement upon delivery of GFP mRNA after ILM photodisruption showing delivery improvement. (iii) Confocal laser scanning microscopy (CLSM) images of the ILM of three human patients disrupted by laser treatment in combination with ICG ex vivo. Adapted with permission from Ref.^175^

For many years, cellular delivery of effector molecules has been explored using viral or non‐viral vectors. In addition, enhancing delivery by cell membrane permeabilization is an alternative or additive approach. This enhancement of membrane permeability can be executed either through chemical methods such as specific solvents or physical techniques via inducing pores in cell membranes.^158^ Of all physical methods, electroporation has been the most assessed but, in this method, therapeutic effectiveness can be attenuated due to cell proliferation and functionality issues.^159^, ^160^ This has led to the introduction of a unique form of intracellular delivery in which photons and PS NPs collaborate concurrently to create pores in cell membrane. The method, namely photoporation, relies on the creation of vapor nanobubbles (VNBs) around the PS NP upon illumination with a pulsed laser. These VNBs are short‐lived and propagate due to a sudden evaporation of water surrounding the NP. The expansion and collapse of these nanoscopic bubbles is a very rapid process which can generate mechanical forces (Figure 5a) and which facilitates pore formation in plasma membranes when the NP is in close proximity to the cells.^161^ In this phenomenon, photoacoustic waves are also generated alongside the formation of VNB but with one major difference compared to when PAI is intended: the laser fluence used for triggering VNBs from the PS is much higher than the excitation capacity of the PS.^162^, ^163^

In the beginning, the formation of VNBs has been explored using plasmonic NPs. Gold nanoparticles were of most interest and research was conducted on different properties by tuning their size and shape. Photoporation was explored to deliver cargo not only to the cytosol of cells, but also at subcellular levels such as the endosomal membrane to promote endosomal escape and even the nuclear envelope to deliver compounds to the nucleus^164^, ^165^, ^166^ (Figure 5b). While great delivery efficiency is observed with plasmonic nanomaterials, their fragmentation upon tremendous laser exposure raises red flags. It has been displayed that fragmented gold NPs can potentially intercalate with DNA and induce genotoxicity.^167^, ^168^, ^169^ To overcome this problem, PDA NPs were developed and successfully used for photoporation because they are biocompatible and biodegradable.^170^, ^171^ However, further studies are required to validate their in vivo degradation process, which is yet not fully understood.^172^ For more elaborate information regarding these PSs and their applications for photoporation, we refer readers to a recent review article published by Xiong et al.^23^

In this context, single small molecules can be promising due to their small molecular structure making their degradation easier for the body. Furthermore, they can broaden the available PS choices. Previous reports of VNB application are only limited to in vitro and ex vivo applications and mainly for photoporation. Most recently, our research group reported the application of these molecules for in vivo applications. They were able to ablate opacities consisting of collagen aggregates formed in the vitreous of the eye (‘eye floaters’) and responsible for vision impairment via ICG clustering at the surface of these opacities and subsequent ablation due to localized mechanical stress provided by the VNBs.^173^ Although our group had previously reported the application of gold NPs for the same purpose,^174^ ICG was used to eliminate the drawbacks associated with gold fragmentation. Besides, ICG is already FDA‐approved for staining the inner limiting membrane (ILM) which covers the retina and is known to have affinity towards certain collagen types (Figure 5ci). Successful ablation of eye floaters in rabbits (Figure 5cii) led to utilizing the same strategy to disrupt the inner limiting membrane (ILM), a major barrier to retinal drug delivery (Figure 5di). After laser irradiation of the previously ICG incubated retinas, a 5‐fold improved delivery of mRNA‐loaded LNPs to bovine retinas was observed (Figure 5dii). To move closer towards clinical translation, ILM disruption of human *ex vivo* specimens were also done successfully (Figure 5diii). However, *in vivo* data is underway and yet to be published.^175^ In addition to using free dyes, ILM photoablation through VNB generation by using dye‐loaded NPs has also been investigated but is seemingly at its early stage.^176^ This finding is important in a since that it demonstrates that dye‐loaded NPs are capable of generating VNBs similar to plasmonic NPs.

### 4.2 Non‐photothermal applications

In this section, activation of the non‐photothermal pathway and its potential benefits through improved fluorescence for FLI and enhanced reactive oxygen species (ROS) generation for PDT are elaborated. ROS are radical species that are generated when oxygen‐containing materials such as oxygen and water interact with an excited PS. The resulting reactive molecules (e.g., hydroxyl (HO•) and superoxide (O_2_•) radicals and singlet oxygen (^1^O_2_)) can eradicate tumor cells through oxidative stress imposed upon cellular components such as lipids, proteins and nucleic acids which is the direct feature of PDT.^177^, ^178^ Furthermore, PDT has shown to indirectly induce immunomodulatory responses by positively affecting dendritic cell (DC) maturation and activation and subsequent T‐cell and B‐cell activation which can also promote cancer therapy.^179^, ^180^, ^181^

#### 4.2.1 Dyes in PDT and FLI

ROS generation improvement has been pursued in various ways. One very common approach is done via chemical alteration of the PS which is done to promote the pathway shift in favor of intersystem crossing (ISC) or enhancing the intramolecular charge transfer (ICT).^40^ The resulting PS will be endowed with improved PDT capabilities and inhibited fluorescence.^182^ With this in mind, Photophrin®, consisting of polymerized porphyrin units, was the first ever FDA‐approved PS for PDT in 1993.^183^ Since then, many other conventional dyes have been chemically modified and tested for PDT. The group of Sun^184^ developed an iodinated cyanine dye (CyI) with enhanced ROS generation capabilities due to the heavy atom effect. It is known that integration of a heavy atom such as high molecular weight iodine into the structure of a PS can enhance spin–orbit coupling resulting in the promotion of the ISC and subsequent improvement of ROS generation capabilities.^185^ This improved ROS generation is accompanied by suppressed FL. However, its translation into clinics was hampered by its poor solubility. To overcome the issue, they fabricated CyI micelles through covalent coupling of HA to the dye via PEG as linker and were even able to prove an immune response as well.^186^

**FIGURE 6:**
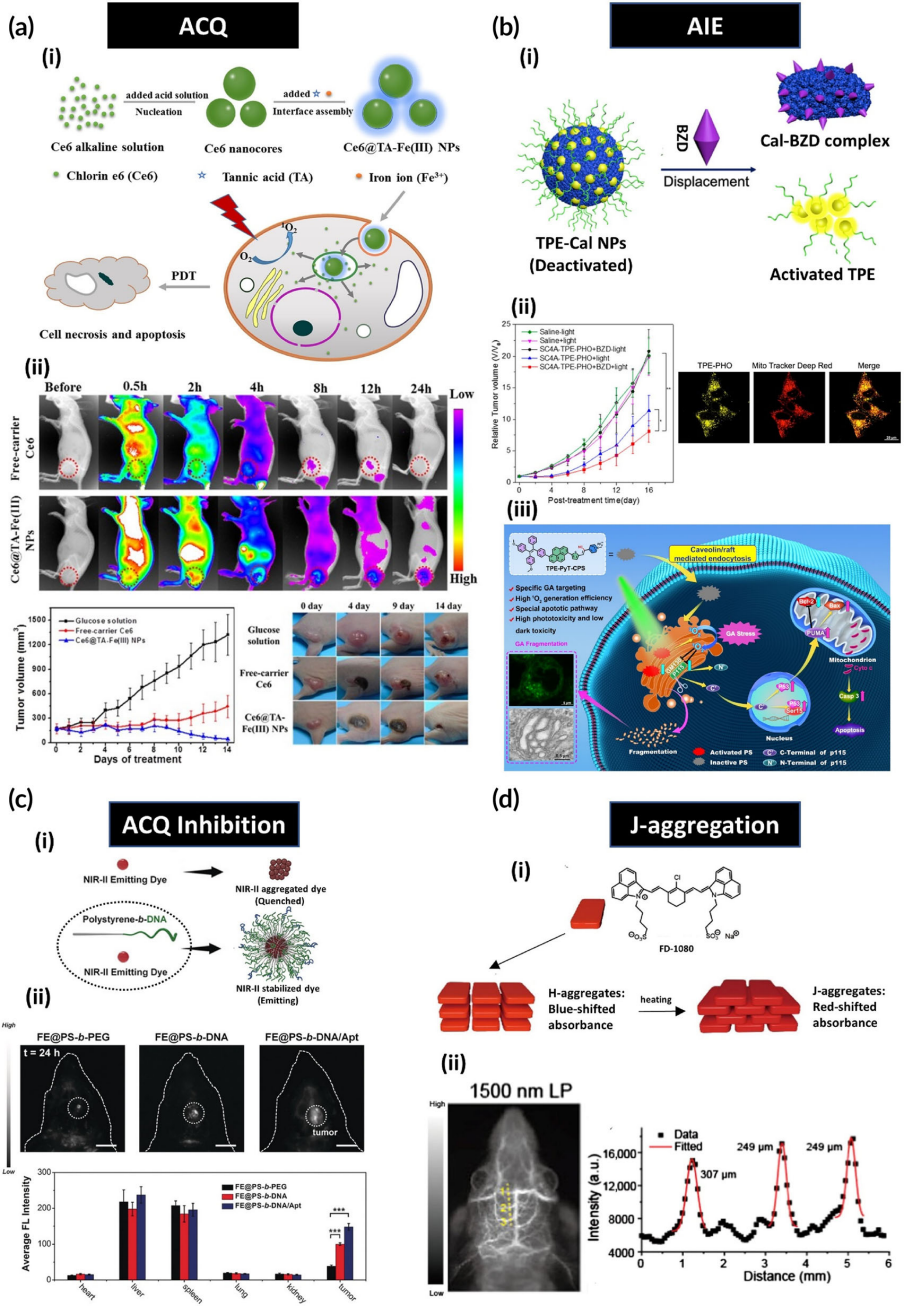
Four different supramolecular engineering methods are exploited for optimal FLI and PDT: (a) Aggregation caused quenching (ACQ) can be exploited to fabricate systems which switch on upon accumulation in cancer cells. (i) Ce6 NP fabrication process and recovery of fluorescence and photodynamic properties upon degradation of NPs inside cells and (ii) fluorescence images and tumor volume suppression data showing photodynamic efficacy. Adapted with permission from Ref.^190^ (b) Aggregation induced emission (AIE) luminogens which become activated upon aggregation in cancer cells. (i) The presence of BZD in the location causes the displacement of TPE and its liberation which then will (ii) activate PDT by accumulation in the mitochondria proven by confocal laser scanning microscopy (CLSM) of TPE inside the organelle. (iii) The proposed mechanism for PDT activity of TPE‐PyT‐CPS inside the golgi organelle: The crosstalk between golgi and mitochondria leads to elevation of caspase‐3 levels and subsequently, induction of apoptosis. (bi) and (bii) are adapted with permission from Ref.^193^ (biii) is adapted with permission from Ref.^196^ (c) ACQ can reduce functionality of NIR‐II dyes therefore (i) by creating hydrophobic pockets provided by NPs (polystyrene in this case), this phenomenon can be inhibited. (ii) In vivo NIR‐II images of brain tumor showing the passage of NPs functionalized with DNA and aptamers through the BBB leading to enhanced penetration and higher contrast images. Adapted with permission from Ref.^214^ (d) Formation of J‐aggregates. (i) FD‐1080 J‐aggregates are formed easily by heating H‐aggregates. (ii) FLI at 1500 nm showing an image in which three vasculatures with two as small as 249 μm are detected. Adapted with permission from Ref.^219^

Although chemical modifications are very effective, they are not always adequate to achieve optimal performance of PSs in biological settings. Many of these compounds are hydrophobic and require a carrier to solubilize and retain them for optimal delivery to the site of action. To address the challenge, a liposomal NP formulation of Verteporfin under the brand name Visudyne® was introduced and is used for the treatment of subfoveal choroidal neovascularization (CNV) due to acute‐macular degeneration (AMD).^187^ Nanoparticle engineering not only provides better delivery of PSs but can also affect their photophysical characteristics which enables researchers to adjust these properties based on the intended therapy. Surprisingly in some cases, supramolecular assembly of PSs can still be a better choice to promote PDT in vitro and in vivo, even if it causes ROS‐generation depletion due to aggregation‐caused quenching (ACQ).^188^ Despite ACQ being known to inhibit PDT efficacy and fluorescence intensity, the reason for better performance of these systems is attributed to a better cellular and tissue distribution of the PS nanoformulations which leads to the recovery of ROS‐generation capacity and fluorescence. For instance, Yan and colleagues demonstrated the successful in vitro application of chlorin (Ce6)‐BSA loaded graphene oxide NPs even though the fluorescence and ROS generation of Ce6 is inhibited in the nanoparticle form. They discovered a more rapid internalization and release of Ce6 from the NPs leading to a recovery of the PDT effect in vitro.^189^ To further prove the applicability of such systems in vivo, they evaluated a novel Ce6 NP formulation which capitalizes on the aggregation tendency of the molecule in neutral aqueous solutions. Upon expansion of the aggregation‐based NPs to the desired size, the process is halted by ultrafast coating with Fe(III) and tannic acid (TA) (Figure 6ai). The obtained Ce6 NPs have quenched fluorescence and ROS generation, however, localization and release from the system in the tumor site recovers their features which results in successful in vivo PDT (Figure 6aii).^190^ Benefiting from the same principle, SQ was quenched by chemically binding the dye to iron oxide NPs using a peptide linker sensitive to cathepsin‐B. Since cathepsin is overexpressed in extracellular matrix (ECM) of triple negative‐breast cancer, the exposure of the system tumor ECM led to enhanced PDT and FL signal enabling in vivo theranostics.^191^

Fortunately, ACQ is not always an observed phenomenon when small molecules are aggregated into NP form. In recent years, the emergence of dyes with aggregation‐induced emission (AIE) properties upon nanoparticle formation has shown promise because of their improved fluorescence and reactive oxygen species (ROS) generation capabilities. Tetraphenylethylene (TPE) and triphenylamine (TPA) AIE luminogens (AIEgens) are among the most typically used dyes for FLI and PDT.^192^ In 2020, Feng et al. developed pyridinium‐functionalized TPA conjugated to calixarene (Cal) NPs and employed them in combination with 4,4′‐benzidine dihydrochloride (BZD) for mitochondria‐specific PDT (Figure 6bi). BZD causes the dissociation of TPE from the TPE‐Cal complex through a competitive displacement process thereafter, the liberated AIEgens translocate from the cytoplasm to the mitochondria. In vivo PDT evaluation showed that both PDT treatments in BZD‐positive and BZD‐negative groups were significant. However, the BZD‐positive group exhibited significantly greater tumor volume suppression than the BZD‐negative group. Additionally, the distribution of TPE inside mitochondria was confirmed by the improvement of fluorescence, which further supports the formation of aggregates inside mitochondria (Figure 6bii).^193^ Golgi apparatus (GA) withholds pivotal role in cellular distribution of proteins inside cells and it is believed that imposing damages to the organelle via oxidative stress can provoke apoptosis.^194^, ^195^ This inspired Guo and coworkers to fabricate TPEs to specifically target golgi organelles in cancer cells for in vivo PDT.^196^ Among their library, TPE‐PyT (л spacer group consisting of pyrene ring and thiophene)‐CPS (cyano‐pyridinium salt) with a D‐π‐A structure exhibited optimal ROS generation and could target golgi organelles very efficiently. This targeting precision was shown to significantly cause in vivo PDT efficiency difference in targeted and non‐targeted formulas. The golgi‐targeted PDT was found to act through the process of crosstalk between ROS‐affected golgi apparatus and the mitochondria, leading to interference with mitochondrial hemostasis and subsequent induction of apoptosis (Figure 6biii). TPA‐based AIEgens have also been explored and proven successful for PDT/FLI or even NIR‐II FLI in vivo.^197^, ^198^

Even though researchers have applied FLI multiple times coupled to other multifunctional modalities, yet again, individual improvement of the modality is a major requirement. Nowadays, fluorophores with absorption and emission in the NIR region are very much of interest since they provide low autofluorescence, less scattering and more penetration depth in vivo.^199^ We have already described the development of dyes with NIR absorption and why they are formulated into NPs in the PAI section. Many of the dyes used in PAI can be used in FLI as well since they undergo radiative energy dissipation. Many examples of in vivo NP‐formulated NIRI dyes exist belonging to the BODIPY,^200^ SQ,^135^, ^201^ phthalocyanine^202^, ^203^ and cyanine^204^, ^205^, ^206^ families. Interestingly, some of these dyes have been investigated for NIR‐II imaging following their emission tail extending as far as 1500 nm. However, it is crucial to expand the library of NIR‐II dyes in parallel, as higher absorption coefficients and quantum yields will result in images with higher resolution in deep organs.^207^

One of the first developed NIR‐II dyes was CH1055, belonging to the BBTD family and introduced by Dai et al.^208^ Following the hydrophobic structure of the dye, the molecules tend to form aggregates which result in quenching leading to a loss of brightness and function. To overcome this drawback, the sulfonated form was conjugated to serum proteins to form supramolecular assemblies which possessed 110‐fold increase in fluorescence. They attributed the fluorescence increase to two factors: inhibition of dye aggregation when coupled to proteins, and geometrical confinement of the dye molecule by hydrophobic protein pockets, which shifts the energy dissipation towards radiative decay. With their system, they were able to image mouse lymph nodes in vivo.^209^ In later works, NIR‐II dye nanoconjugates were synthesized under the same principle, namely the D‐A‐D structure, with the inclusion of hydrophobic pockets to reduce the possibility of ACQ. This resulted in higher quantum yields, enabling in vivo imaging of brain injuries and vasculature maps.^210^, ^211^ Based on the evidence that polystyrene has been shown to be an effective hydrophobic pocket for dye loading,^211^, ^212^, ^213^ the Tian research group^214^ fabricated nanoparticles (NPs) composed of a polystyrene core that hosted the NIR‐II fluorophore, and a dense exterior of DNA strands that allowed the NPs to cross the blood–brain barrier and image in vivo (Figure 6ci). Furthermore, surrounding packed DNA was utilized to hybridize aptamers for glioblastoma targeting. Overall, the presence of DNA in their carrier provided 3.8‐fold fluorescence intensity in vivo compared to the counterpart without the DNA and with PEG instead (Figure 6cii).

In contrast to enhancing FLI performance by inhibiting self‐aggregation resulting in quenching, some dyes show improved FLI properties upon self‐aggregation. AIEgens and J‐aggregates are two of the kind. Despite the fact that in most cases, aggregation reduces radiative decay by quenching resulting in lower fluorescence, J‐aggregates have proven worthy for application in fluorescence imaging by improving absorption towards NIR, absorptivity and in some cases even fluorescence quantum yield.^215^, ^216^ We have already given some examples of AIEgens thus now we will elucidate the recent advances in J‐aggregation.

Most reported J‐aggregates are emissive in the visible to NIR‐I window whereas NIR‐II emissive J‐aggregate are limited. In addition, most of these NIR‐II molecules have absorption maxima limited to the NIR‐I region. Therefore, developing molecules and systems that possess both excitation (Ex) and emission (Em) in the NIR‐II region is of great value. Slatten and coworkers were among the first to develop cyanine dye J‐aggregates of IR‐140 inside pores of mesoporous silica NPs and obtain Ex/Em wavelengths of 1042/1043 nm merely by changing the solvent during preparation.^217^ Similarly, the group of Zhang formulated J‐aggregates of another cyanine candidate, FD‐1080 by utilizing the 1,2‐dimyristoyl‐sn‐glycerol‐3‐phosphocholine (DMPC) lipid. They were able to achieve Ex/Em wavelengths of both ~1300 nm and conducted in vivo FLI in the emission tail of 1500 nm wavelength by 1064 nm excitation laser.^218^ However, encapsulation efficiency and size distribution of obtained nanoscopic aggregates were not optimal therefore, these J‐aggregates were fabricated basically from heating H‐aggregates of FD‐1080 to overcome the issues of the previous experiments (Figure 6di). The optimally obtained J‐aggregates were then tested in vivo and FLI was implemented on mice brain vasculature with the same setting as their previous study. Benefiting from the principal component analysis (PCA) of the dynamic imaging of the area, discrimination between arteries and veins was attained together with cerebral vasculature width measurements as low as 249 μm (Figure 6dii).^219^ Compared to other examples of NIR‐II FLI utilization in which natural (e.g., albumin)^220^ and synthetic polypeptides (e.g., PEA)^221^ were used as scaffold for J‐aggregation, this facile one pot synthesis of the NIR‐II agent is more promising for clinical translation. Other potential candidates are from the BODIPY family. These NIR‐II agents are only NIR‐II emissive and obtained by means of J‐aggregation. Evidently, chemical grafting of donor and acceptor groups have shifted their FL emission to NIR‐II window,^222^, ^223^ nevertheless, these molecules are scarce and outperformed by cyanine candidates in NIR‐II FLI.

#### 4.2.2 Dyes as stabilizers

As explained previously, similar to many hydrophobic molecules, dyes have the propensity to form aggregates in aqueous media. Throughout this review, we brought up the imposed limitations and benefits regarding the photophysical properties that are associated with aggregation. In recent years, a new field has emerged where researchers exploit the hydrophobicity and aggregation tendency of dyes to form stable colloidal aggregates than can accommodate and stabilize proteins and drugs. The group of Shoichet investigated the stabilization of seven different aggregator drugs using evan blue (EB) and congo red (CR) dyes as stabilizers.^224^ Interestingly, application of these dyes yielded <100 nm NP batches with homogenous size distributions. Furthermore, it was shown that these NPs can bind to enzymes. As a result of their interaction, enzymatic activity was inhibited whereas activity recovery was observed upon release from the NP. Further studies on CR‐stabilized particles revealed their preferential affinity towards proteins in comparison to nucleic acids and even peptides that constituted the studied protein.^225^ In a later study, by the aid of accurate and quantitative calculations, Shamay and coworkers were able to employ IR783 as a sulfated indocyanine that has potential drug stabilizing properties to stabilize hydrophobic drugs such as sorafenib and trametinib.^226^ These drugs are known to pharmacologically exhibit anticancer properties through the inhibition of tyrosine kinase. While consequent inhibition of extracellular signal‐regulated kinases (ERK) phosphorylation associated with the inhibition of this enzyme can cause skin rashes, stabilization of these drugs using dyes removed this side effect since release of the drug in the tissue is avoided. Besides, it was found that these nanocarriers can selectively target cancer tissue through caveolin‐1‐mediated endocytosis in a genetically modified mouse model for hepatocellular carcinoma and a xenograft model for human colorectal cancer. An interesting approach they implemented in their studies was the use of the computational method of quantitative structure property relationship (QSPR). Firstly, IR783 was screened together experimentally with 16 other hydrophobic drugs, 4 of which were shown to be able to generate NPs. Thereafter, by using the computational model, approximately 5000 molecular descriptors that were deemed to be important indicators for nanoassembly formation, were analyzed by software with only four of them having a high correlation with the experimental NP formation. This finding highlights the ability of computational models to filter between numerous set of molecules and choose the best possible candidates. In a later work, the applicability of such particles were demonstrated by using IR783 stabilized sorafenib in combination with electroporation to induce changes in tumor vasculature and microenvironment. This yielded a 40% reduction in tumor volume of mice bearing colorectal tumors.^227^ The promising results with the sulfated cyanine dyes and computational predictive methods for dye stabilizer identification inspired Shamay et al. to explore an automated approach in which synthesis and testing of drug stabilizers were carried out using automated machines.^228^ Using some of the original building blocks of dyes such as IR783 and IR820 together with other novel precursors, multiple synthesized dyes were screened in order to choose the best stabilizing component. In parallel, the aggregator drugs were also monitored based on their AIE properties and trametinib, a kinase inhibitor, was the chosen drug. The stabilization process was screened in an automated fashion and based on analyzing the extent to which the synthesized stabilizer inhibited the AIE of the drug component. Interestingly, two novel compounds were identified with R595 having superior stabilization property. This promising molecule resembles polydopamine and is easily scalable. However, further studies are required to evaluate the NP formation of R595 with other hydrophobic compounds with different degrees of aromatic rings and π‐conjugated groups as in the current paper, molecules with a certain chemical pattern were studied. Similarly, and in a different work, the same group screened the NP formation capability of 77 different cancer drug pairs that are known to be biologically synergistic by analyzing their AIE inhibition after NP formation. This analysis led to classification of these drugs into five different categories. Then, based on the classification, they were able to develop a machine learning model that is able to predict any stable drug–drug self‐assembly defining them as meta‐synergistic drug pairs due to their chemical as well as biological synergies.^229^

## 5 CONCLUSION AND PERSPECTIVES

In the past few decades, PSs have shown promise in a variety of applications from fluorescence and photoacoustic imaging to photothermal and photodynamic therapies. Plasmonic and carbon‐based nanomaterials are two types of agents which have been heavily investigated. Despite their ability to react to light, their biocompatibility and biodegradability have been in question.^26^, ^27^, ^167^, ^168^, ^169^ Therefore, small organic dyes have shown growing interest and strong promise. Thanks to their versatile chemical moieties and their ability for chemical modification, their degradability can be improved and their photophysical properties can be more easily adjusted.

Regardless of the typical advantages associated with supramolecular nanoassembly of dyes, namely improved accumulation and body distribution, the use of dyes in this form has been shown to significantly improve the photosensitization yield of these molecules. Seemingly, this finetuning is achieved by improvement of their photophysical properties upon nanoformulation. To clarify this, in our review, we have described instances where dyes are driven towards forming certain aggregation types such as J and H aggregation to improve the photophysical characteristics or stabilization to avoid the ACQ phenomenon. In addition, AIE luminogens are introduced. These molecules emit light when they aggregate, which underlies the importance of their encapsulation and/or formation into nanoparticle forms. Recently, there has been a growing interest in developing NIR absorbing and/or emitting PSs due to their superior penetration depth. These molecules typically have relatively large conjugated systems in terms of chemical structure, which makes them susceptible to aggregation and can lead to decreased activity. Formation of these molecules into NPs can be a good strategy to address this limitation by either preventing aggregation or directing it towards a specific form.

Based on the current trend regarding the joint applications of dyes and nanoparticles, it is evident that systems designated for PAI and PTT require high absorption yields and PCEs to ensure optimal performance. In addition, to further realize the feasibility of the in vivo application, molecular and supramolecular design is guided towards achieving systems with absorption in the NIR‐I and NIR‐II windows which then enables deep tissue diagnosis and therapy. The principles are similar for non‐photothermal applications such as FLI and PDT with few important points: (a) for both modalities, PCE is hampered to direct the energy dissipation towards light emission and photodynamic enhancement and (b) when FLI is intended, in addition to the incident light, there is the emitted light that is produced by the PS and is required to reach the detector; therefore, it should have enough brightness and photostability to avoid bleaching.

While previously mentioned modalities have been heavily studied and reviewed, we introduced the generation of VNBs to perform photoablation of biological aggregates or barriers as a novel application. The latter is based on the photothermal properties of dyes and benefits from the mechanical forces generated by the collapse of VNBs following the excitation of dye clusters with a pulsed‐laser. Currently, in vivo applications of this modality are limited to ophthalmology for the removal of vitreous opacities (”eye floaters”) and disruption of the internal limiting membrane (ILM) to improve the delivery of drugs into the retina.^173^, ^175^ However, future applications in other organs, such as, for example, in the brain for the elimination of protein aggregates (e.g., α‐synuclein and β‐amyloid), which play a role in Parkinson's and Alzheimer's disease, may also hold promise.^230^

Finally, application of dyes is not only limited to their photophysical characteristics. These molecules can also contribute to stabilization of drugs and in some cases, the capture and release of proteins. This field is very promising in which single small molecules are exploited to identify other stabilizers or contribute to NP formation as stabilizers themselves resulting in high formulation stability and encapsulation yield. When dyes are used as stabilizers, their interaction with drugs and proteins is deemed to be through π–π and hydrophobic interactions. Dyes such as congo red and IR783 were discovered to possess such stabilization ability. However, exploring other dyes with Ex/Em wavelengths shifting further towards the NIR‐II region, with the intention of providing superior imaging capabilities as well as therapy, could be a next milestone to reach.

## AUTHOR CONTRIBUTIONS

**Pouria Ramezani:** Conceptualization (equal); writing – original draft (lead); writing – review and editing (supporting). **Stefaan C. De Smedt:** Funding acquisition (equal); project administration (equal); writing – review and editing (supporting). **F**é**lix Sauvage:** Conceptualization (equal); funding acquisition (equal); project administration (equal); writing – review and editing (lead).

## CONFLICT OF INTEREST STATEMENT

The authors declare no conflict of interest.

### PEER REVIEW

The peer review history for this article is available at https://www.webofscience.com/api/gateway/wos/peer-review/10.1002/btm2.10652.

## Data Availability

No new data were generated or analyzed for this manuscript.
